# Role of monocytes/macrophages in renin-angiotensin system-induced hypertension and end organ damage

**DOI:** 10.3389/fphys.2023.1199934

**Published:** 2023-10-03

**Authors:** Tlili Barhoumi, Stephen Todryk

**Affiliations:** ^1^ Medical Research Core Facility and Platforms (MRCFP), King Abdullah International Medical Research Center, King Saud Bin Abdulaziz University for Health Sciences (KSAU-HS), Riyadh, Saudi Arabia; ^2^ King Saud Bin Abdulaziz University for Health Sciences, Riyadh, Kingdom of Saudi Arabia; ^3^ Department of Applied Sciences, Northumbria University, Newcastle Upon Tyne, United Kingdom

**Keywords:** monocytes, macrophages, renin-angiotensin system, hypertension, innate immunity

## Abstract

The renin-angiotensin system (RAS) is a central modulator of cardiovascular physiology. Pathophysiology of hypertension is commonly accompanied by hyper-activation of RAS. Angiotensin II receptor blockers (ARBs) and Angiotensin-converting enzyme (ACE) inhibitors are the gold standard treatment for hypertension. Recently, several studies highlighted the crucial role of immune system in hypertension. Angiotensin-II-induced hypertension is associated with low grade inflammation characterized by innate and adaptive immune system dysfunction. Throughout the progression of hypertension, monocyte/macrophage cells appear to have a crucial role in vascular inflammation and interaction with the arterial wall. Since myelomonocytic cells potentially play a key role in angiotensin-II-induced hypertension and organ damage, pharmacological targeting of RAS components in monocyte/macrophages may possibly present an innovative strategy for treatment of hypertension and related pathology.

## Graphical Abstract

**Figure:**
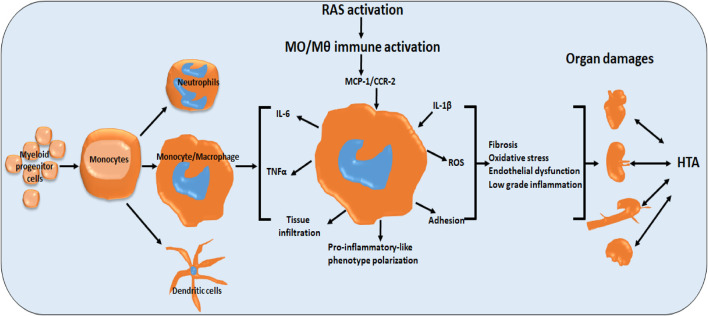
Role of monocytes/macrophages in renin-angiotensin system-induced hypertension and end organ damage. RAS activation induces monocyte/macrophage pro-inflammatory-like phenotype polarization, directly and indirectly via cytokines (MCP-1, CCR2, IL-1b). Activation manifests in fibrosis, oxidative stress, endothelial dysfunction and low grade inflammation (IL-6, TNFa) and tus end organ damage. (HTA hypertension).

## Introduction

More than 1 billion people worldwide are affected by hypertension (Collaboration, 2021). Hypertension, a “silent killer”, is the major cause of cardiovascular diseases, and is estimated to cause more than 7 million deaths globally per year. The renin-angiotensin system (RAS) plays a pivotal role in regulation of cardiovascular diseases including hypertension. Hyper-activation of RAS components is commonly accompanied by dysregulation of blood pressure and associated inflammation. Immune system and inflammation are emerging as being involved in hypertension and vascular diseases (Wenzel, 2019). Angiotensin-II-induced hypertension and end organ damage is characterized by abnormality of myelomonocytic cell function and significant infiltration of monocyte/macrophages into heart, kidney, aorta and brain. Monocyte/macrophages express different RAS elements such as Angiotensin II receptors (ATR) and angiotensin-converting enzymes (ACE1/2). The presence of ACE as an intracellular component or released by activated cells highlights its local or systemic role as a modulator of inflammatory immune responses (Song et al., 2015)during hypertension. Yet, the use of ACE inhibitors as one of the standard treatments for elevated blood pressure may shed light on the role of this enzyme, specifically in monocyte/macrophages in the course of hypertension. The characteristics of plasticity in monocyte/macrophages, the RAS components present in these cells, as well as the emerging role of local RAS and counter-regulatory RAS, are the major aspects in this area that still not fully understood.

### Renin-angiotensin system: Role in inflammatory immune responses associated with hypertension

Renin-angiotensin system (RAS) has two major axes. The classical axis ACE/Ang II/angiotensin II type 1 receptor (AT1R), and the counter-regulatory axes ACE-2/Angiotensin 1–7 (Ang 1–7) Mas receptor (MasR) and ACE2/Ang 1–9/AT2R (Sepulveda-Fragoso et al., 2021; MacLachlan et al., 2023). The Mas axis was identified recently as a factor involved in macrophage function during inflammatory processes in vascular and central nervous systems (Hammer et al., 2016). Ang-(1–7) play the role of anti-inflammatory factor through polarization of macrophage toward the M2 like-phenotype (Pan et al., 2021). Both RAS pathways are dependent on ACE and ACE-2 (Sharma et al., 2023). It was reported that inflammation and vascular and end organ damage observed during hypertension are mediated by vasoconstrictor components such as endotheline-1 (ET-1) (Li et al., 2013) or RAS activation which modulates immune responses. We and others reported previously that T cell play a crucial role in inflammation associated with development and progression of hypertension (Schiffrin, 2021; Caillon et al., 2019). More deeply we shown that Treg cells are directly implicated in angiotensin II induced hypertension (Barhoumi et al., 2011) and vascular damage and that γδ T cells mediate Ang II-induced SBP elevation, vascular injury, and T-cell activation (Caillon et al., 2017). More recently we shown that Ang II triggered functional polarization of Tcell subpopulations, by increased release of pro-inflammatory IFN-γ, TNF-α and IL-17, toward CD4^+^ Th1/Th17 and CD8^+^ Th17 inflammatory-like-phenotypes (Almutlaq et al., 2022). Yet, activation of Ang II might be counteracted by Ang III and IV which are the end product of Ang II cleaved by endoplasmic reticulum aminopeptidase 1 and 2 (ERAP1 and ERAP2). ERAP1 and ERAP2 are essential for the generation of major histocompatibility complex (MHC) class I binding peptides (Compagnone et al., 2019) and innate immune responses (Saulle et al., 2020; Blake et al., 2022). Presence of RAS components throughout the body from organs to tissues, as well as cells, define the role of local RAS systems and ACE function. However, RAS components like Ang-I, Ang-II, AT1 and AT2 receptors, ACE1, ACE2 and renin, are differentially expressed in all immune cells such as monocyte/macrophage (Okamura et al., 1999). Recently, emergence of both adaptive and innate immune system in pathogenesis of hypertension become more evident (Hengel et al., 2022). Throughout inflammatory processes, adaptive and innate immune responses may be stimulated by the activation of the ACE/Ang II/AT1R axis, leading to cardiovascular damage and autoimmune disorders (Table 1). Furthermore, activation of the ACE/Ang II/AT1R axis is regularly associated with apoptosis and proinflammatory cytokine release as well as neutrophil and macrophage chemotaxis associated with organ injury (Almutlaq et al., 2021).

**TABLE 1:** Summary of major role of RAS components in innate/adaptive immune system.

RAS components	Role in innate/adaptive immune system
Angiotensin II (Ang II)	stimulates the innate immune responses via activation of Toll-like receptor 4 (TLR4) PMID: 28330785
	local production of angiotensin II regulates T cell function PMID: 19073907
	promote M1 macrophage polarization PMID: 34514000, PMID: 32186758
Ang-(1–7)/MasR	anti-inflammatory and anti-fibrotic processes PMID:27649628
	migration of monocytes/macrophages and phagocytosis PMID: 34874920
	inhibited inflammatory responses *in vivo* and *in vitro* and promoted M2 phenotype PMID: 34045880
Angiotensin converting enzyme ACE	ACE overexpression can affect innate and adaptive immunity. PMID: 17525278
	Overexpression in macrophages drives antitumor and antimicrobial responses PMID: 33583391
	in antigen presenting cells, ACE participates in regulation of both major histocompatibility complex (MHC) class I and MHC class II PMID: 29578208
Angiotensin type 1 (AT1R)	Angiotensin type 1 receptor modulates macrophage polarization toward M1-like-phenotype PMID: 21367915, PMID: 24743144

ACE-2 plays a crucial role in cleaving Ang II to Ang 1–7. Ang 1-7 cleaved by ACE-2 from Ang II counteracts the deleterious effects of the activated RAS and play a protective role in control blood pressure-rise (Fontes et al., 1994). During inflammation, ACE-2/Ang 1-7 axis protects vascular dysfunction by inhibition of vascular cell adhesion protein 1 (VCAM-1), monocyte chemoattractant protein-1 (MCP-1) and signatures of pro-inflammatory monocyte/macrophage such as interleukin-6 (IL-6), tumor necrosis factor alpha (TNF-α) and reactive oxygen species (Lelis et al., 2019). Whilst Ang 1-7 is a product of Ang II via ACE-2, it can additionally be induced from Ang 1-9 cleaved by ACE. Furthermore, ACE induces degradation of Ang 1-9 to Ang 1-7 and ACE-2 cleaves Ang I to Ang 1–9. This loop includes ACE/ACE-2/Ang 1–7/Ang 1–9 as protective mediators serving to counterbalance the pro-inflammatory effects of activated RAS and ameliorating vascular damage and arterial hypertension, induced (Mendoza-Torres et al., 2018) mainly via prevention of oxidative stress and downregulation of IL-6, IL-1β, MCP-1, and TNF-α released mostly by monocytes/macrophage (Cha et al., 2018).

### RAS-induced hypertension modulates monocyte/macrophage function and immune responses

Circulating monocytes with different phenotypes indicate a clear status of the systemic immune functions which reflect the effects of infection and inflammatory responses on severity and possibly lethal complications (Wang et al., 2023). Activation of paracrine or endocrine signaling processes modulate differentiation and polarization of monocytes. Monocyte/macrophage express ATR (Hahn et al., 1994), angiotensinogen (Gomez et al., 1993), renin (Iwai et al., 1996), as well as angiotensin peptide hormone (Dezso et al., 1989) thus, monocyte/macrophage activity and function is modulated by RAS components during hypertension. ACE and ACE2 are differentially expressed in monocyte/macrophages from blood or tissues, and for that reason, up- or downregulation of ACE/ACE2 in Monocyte/macrophages may modify the function and the immune reaction specifically as it was reported that antigenic or mitogenic stimulation may regulate ACE/ACE2 expression in macrophages (Covian et al., 2020). Additionally, it was reported that the differentiation of monocytic THP-1 cells to macrophage-like-phenotype is associated with upregulation of intracellular and released RAS components in these cells (Okamura et al., 1999).

The role of inflammation in vascular injury was extensively studied in atherosclerosis, including infiltration of leucocytes and monocyte/macrophage differentiation, as well as cytokine release and systemic and local innate immune responses (Mallat and Binder, 2022; Gusev and Sarapultsev, 2023). These investigations stimulate new studies to evaluate the role of inflammation in vascular damage-inducing hypertension. We and others reported that B and T cells are implicated in Ang II-induced hypertension and vascular dysfunction (Barhoumi et al., 2011; Guzik et al., 2007). The implication of inflammatory mechanisms in hypertension was demonstrated in different hypertensive animal model studies such as infiltration of monocytes/macrophage into vascular wall of heart, kidney, and brain. Infiltration of monocytes into target organs is usually a consequence of renin-angiotensin-aldosterone system (RAAS) activation facilitated by the presence of angiotensin receptors (ATR) in almost all cells, tissues and organs. These observations explain the variation of the immune profile of splenocytes shifting to pro-inflammatory status in Ang-II-induced hypertension, and the decline following treatments. Interestingly, hypertensive therapeutic interventions commonly provide a significant reduction in monocyte/macrophage infiltration and expression (Hilgers et al., 2000).

Endothelial dysfunction, oxidative stress and inflammation associated with hypertension induced by Ang-II or DOCA/salt, are prevented in models deficient in macrophage colony stimulating factor (M-CSF or CSF-1) inducing decrease of monocyte/macrophage cell profile (De Ciuceis et al., 2005; Ko et al., 2007). Additionally, depletion of macrophages in rats reduced blood pressure (Thang et al., 2015). To investigate in detail the role of innate immune system in hypertension and vascular disease we previously tested another model using endotheline-1 (ET-1), a potent vasoconstrictor released by vascular endothelial cells, and stimulator of hypertension and vascular inflammation. We found that reduced macrophage-dependent inflammation improves endothelin-1-induced vascular injury (Javeshghani et al., 2013).

Due to the difficulty to study organ macrophage infiltration in hypertensive patients, most human studies focus on peripheral blood monocytes. Circulating monocytes are activated and display more adherence to endothelial cells during hypertension (Chen et al., 1999; Dorffel et al., 1999) thus, exhibit a pro-inflammatory-like-phenotype via release of interleukin (IL)-1 and tumour necrosis factor-α and transforming growth factor-β (Porreca et al., 1997). Activation of the ACE/Ang II/AT1R axis induces endothelial dysfunction and vascular remodeling specifically in small vasculature (resistance arteries) which are the key step in development of vascular resistance, a typical feature of hypertension.

Adhesion of monocytes to the endothelium is a signature of vascular inflammation (Gonzalez-Granado et al., 2023). Ang-II induces an increase in endothelial vascular cell adhesion molecule 1 (VCAM‐1) and intercellular adhesion molecule‐1 (ICAM‐1) as well as E-selectin and P‐selectin. Ang-II binding to AT1R and AT2R stimulates adhesion molecules expression on endothelial cells and trigger ligands on monocytes to promote cell attachment. Angiotensin-II increases monocytes adherence to the endothelium via the MCP‐1/CCR‐2 axis (Mateo et al., 2006; Abu Nabah et al., 2007). Yet, myelomonocytic cells and monocytes attached to the vascular endothelium release pro-inflammatory cytokines such as IL-6 which is independently associated with hypertension (Bautista et al., 2005). In the other hand, monocytes may be also activated by vascular endothelial cells. Loperena et al., investigated the interaction between aortic vascular endothelium and monocytes in Ang-II-induced hypertension. They reported a change in monocyte phenotype and conversion toward more pro-inflammatory cells (CD14^++^CD16^+^ intermediate) with increase in expression of IL-23, IL-1β and TNFα. Interestingly, the activation of monocytes is stimulated by endothelial dysfunction probably due to Ang-II induced ROS generation and IL-6 release and also due to monocyte STAT activation (Loperena et al., 2018). Furthermore, genetic depletion of IL-6 prevents angiotensin II-induced hypertension (Lee et al., 2006) indicating that control of angiotensin-induced hypertension may be modulated by preventing monocyte vascular adhesion and fine tuning of IL-6 release and signaling pathway activation. Furthermore, Wenzel et al., 2015 (Wenzel et al., 2015) reported that heme oxygenase-1 (HO-1) which is a modulator of endothelial function in Ang-II-induced hypertension and vascular diseases suppresses the pro-inflammatory phenotype of monocytes/macrophages and controls their function in Ang-II induced hypertension animal model and human.

### Role of monocyte/macrophage in RAS-induced-hypertension and target organ damages

Vasculature: It is well documented that abnormal activation of RAS stimulates vasculature as primitive end-organ damage by triggering endothelial dysfunction, oxidative stress and vascular remodeling which increase blood pressure (Nangaku and Fujita, 2008). However, in the last decade it has become more accepted that inflammation is a major element involved in hypertension and that hyperactivation of RAS is associated with low grade inflammation inducing hypertension (Rizzoni et al., 2022). The inflammation process in hypertension is mediated by monocyte/macrophage cells as a part of innate immune responses, including bone marrow-derived cells circulating in blood vessels, and myelomonocytic cells and macrophages infiltrating different organs and tissues specifically during angiotensin-induced hypertension (Mian et al., 2014). Dysfunction and local inflammation of small arteries is a window for detection of RAS dysregulation related to hypertension and end-organ damage. Infiltration of monocyte/macrophages through the endothelium, and differentiation to resident macrophages, plays a crucial role in low-grade inflammation-associated to hypertension.

We recently demonstrated that Ly6C^hi^ and CD11b^hi^ monocytes and macrophages are also highly expressed and activated in perivascular tissue in Ang-II-induced hypertension (Barhoumi et al., 2017). Importantly, these effects are mediated by cellular interaction with other components such as matrix metalloproteinase-2 (MMP2) which is necessary for circulatory immune cells activation. In this context we used *Mmp2* knockout mice and we found that all Ang-II effects were blunted (Barhoumi et al., 2017). Monocytes and resident macrophages play a dual role throughout the inflammatory process and homeostasis, depending on their polarization as pro-inflammatory monocytes in the form of Ly6C^hi^ and Ly6C^lo^ as anti-inflammatory as well as M1 pro-inflammatory or M2 anti-inflammatory macrophage-like phenotypes. It was demonstrated that preactivation of circulatory hypertensive patients’ monocytes leads to a high level of IL-1β pro-inflammatory cytokine release (Dorffel et al., 1999) and that *in vitro* stimulation of monocytes isolated from spontaneous hypertensive rats had increased secretion and expression of TNFα and IL-1β (Liu et al., 1996). Furthermore, human peripheral monocytes may be activated by Ang-II (Hahn et al., 1994) and treatment with angiotensin receptor blocker (ARB) may decrease interleukin-1β release (Li et al., 2005).

Interaction between circulating monocytes and the endothelium is crucial for local innate immune responses, and regulation of such interaction mediates the differentiation and function of myeloid cells during inflammation. Hypertension is frequently accompanied by endothelium activation which in turn stimulates blood cell function and differentiation (Loperena et al., 2018). Recently, the Harrison group investigated the role of endothelium/monocyte interaction in hypertension. They suggested that endothelial dysfunction leads to oxidative stress, and pro-inflammatory IL-6 cytokine release as well as disturbance of nitric oxide (NO) signaling may have a major role in monocyte intracellular STAT3 activation (Loperena et al., 2018).

Pathogenesis of atherosclerosis is similar to that found in different vascular damages associated with angiotensin II-induced hypertension. More specifically the role of myelomonocytic cells in hypertension and Ang-II-induced vascular damages has been recently investigated. It was reported that Lysozyme M-positive (LyzM+) monocytes regulates Ang-II-induced hypertension and vascular injury (Wenzel et al., 2011). Depletion of LyzM + prevents endothelial and vascular smooth muscle cell dysfunction and reduces arterial ROS production. However, adoptive transfer of wild-type CD11b^+^Gr-1^+^ monocytes rescue the effect of Ang-II, whereas adoptive transfer of CD11b^+^Gr-1^+^ neutrophils or monocytes from mice lacking ATR1 receptor has no effect, indicating the role of local RAS component expression in monocyte in regulating their function.

Kidney: Renin, as a major component of RAS, is mostly released by kidneys. Dysfunction of kidney is related to dysregulation of electrolyte balance and blood volume control associated usually with blood pressure rise (Wenzel, 2019; Goldblatt, 1947) and more advanced inflammation-induced hypertension and kidney damage. It is well known that inflammation is a signature of kidney dysfunction related to hypertension (McMaster et al., 2015). Nevertheless, inflammation and immune responses within kidney compartments are still under investigation as potential precursors of hypertension. Ang-II leads to a permanent infiltration of myelomonocyte in the renal compartments (Ozawa et al., 2007). We and others (Ozawa et al., 2007) reported that Ang-II infusion induced T cell infiltration as well as monocyte/macrophages into the medulla and renal cortex. More recently, it was demonstrated also that Ang-II stimulates differentiation of renal myeloid cells toward macrophage-like-phenotype (Ly6^C+^Ly6^G−^) (Zhang et al., 2016) and that differentiation is prevented by IL-1 receptor (IL-1R1) activation. The role of IL-1 in renal macrophages was reported by the same group as a pro-inflammatory cytokine released by kidney macrophages in a RAS-mediated hypertension model (Crowley et al., 2010). Chemokine/cytokine release and production are crucial for cell homeostasis and immune function and responses. They control cell-cell interaction, proliferation and migration; thus their expression is differentially regulated in myeloid cells after activation to modulate adequate immune responses (Kopydlowski et al., 1999). Additionally, it was demonstrated that in absence of chemokine chemokine (C-C motif) ligand 5 (CCL5) mice presented a status of kidney damage characterized by tissue infiltration of macrophages with pro-inflammatory signatures. Furthermore, CCL5 prevented monocyte/macrophages accumulation in Ang-II induced renal injuries through inhibition of CCL2 pro-inflammatory effects (Rudemiller et al., 2016). During RAS activation, Ang-II induced recruitment of inflammatory cells via NF-kappaB activation and synthesis of MCP-1 that is blunted by ACE inhibitor treatment (Ruiz-Ortega et al., 1998) which explains at least in part the advantageous effects in kidney diseases.

Heart: Angiotensin II (AngII) signaling and effects are modulated mainly through the ATR1 and ATR2. The presence of ATR in different tissues and organs such as heart, kidney, aorta and brain explain end-organ injury during RAS activation. We previously demonstrated that 2 weeks of infusion with angiotensin II in mice induced hypertension and cardiac hypertrophy associated with fibrosis and massive monocyte/macrophage infiltration. Local infiltration of myeloid cells mediated partially Ang-II-induced cardiac cells proliferation such as myocytes (McEwan et al., 1998), fibroblasts, as well as myelomonocytic cells (Muller et al., 2000). Prevention of low-grade inflammation by increasing the number of Treg cells (Kvakan et al., 2009) or by inhibition of IFN-γ (Marko et al., 2012), reduced cardiac immune cell infiltration in the form of T cells and monocyte/macrophages.

Brain: The synthesis of RAS components by nervous system cells point out the possible relevant role of this system and suggest that the interaction with glia and neuron may regulate blood pressure in brain diseases (de Kloet et al., 2015). It was reported also that dysregulation of RAS in brain exacerbate oxidative stress and activate neurodegeneration in elderly (Labandeira-Garcia et al., 2017). Recently, it was suggested that inhibition of ACE/Ang II/AT1 axis or activation of ACE/Ang II/AT1 axis is considered as a potential target for treatment of neurological diseases (de Miranda et al., 2022).

## Conclusion

Inflammation and innate immunity play a major role on angiotensin-II-induced hypertension and vascular damage. Activation of RAS components expressed in monocyte/macrophage modulates cell function and interaction with vascular endothelium. Hyper-activation of ACE/Ang II/AT1 axis or dysregulation of ACE-2/Angiotensin 1–7 (Ang 1–7) MasR axis potentially aggravates endothelial dysfunction, fibrosis, oxidative stress, monocyte/macrophage cell infiltration to perivascular tissue, in the kidney and heart, leading to blood pressure rise. Nevertheless, the signaling pathways by which angiotensin-II interacts with the monocyte/macrophage cells during hypertension and the specific role of ACE and ACE-2 remain unrevealed, hence more studies are necessary to clarify the mechanisms of Ang-II activating monocyte-macrophage cells during hypertension, and to highlight potential target treatments.

## Abbreviations

RAS, renin-angiotensin system; ARBs, Angiotensin II receptor blockers; ACE, Angiotensin-converting enzyme; ATR, Angiotensin II receptors; Ang 1–7, Angiotensin 1–7; MasR, Mas receptor; RAAS, renin-angiotensin-aldosterone system; M-CSF, macrophage colony stimulating factor; VCAM‐1, adhesion molecule 1; ICAM‐1, intercellular adhesion molecule‐1; ET-1, endothelin-1.
